# A New Data Repository for Pharmacokinetic Natural Product-Drug Interactions: From Chemical Characterization to Clinical Studies[Fn FN3]

**DOI:** 10.1124/dmd.120.000054

**Published:** 2020-10

**Authors:** Caroline Birer-Williams, Brandon T. Gufford, Eric Chou, Marijanel Alilio, Sidney VanAlstine, Rachael E. Morley, Jeannine S. McCune, Mary F. Paine, Richard D. Boyce

**Affiliations:** Department of Biomedical Informatics (C.B.-W., E.C., R.D.B.) and School of Pharmacy (M.A.), University of Pittsburgh, School of Medicine, Pittsburgh, Pennsylvania; School of Pharmacy, University of Utah, Salt Lake City, Utah (S.V., R.E.M.); Covance Inc., Clinical Pharmacology, Madison, Wisconsin (B.T.G.); Department of Population Sciences and Department of Hematology & HCT, City of Hope Comprehensive Cancer Center, Duarte, California (J.S.M.); Department of Pharmaceutical Sciences, College of Pharmacy and Pharmaceutical Sciences, Washington State University, Spokane, Washington (M.F.P.); and Center of Excellence for Natural Product Drug Interaction Research, Spokane, Washington (J.S.M., M.F.P., R.D.B.)

## Abstract

There are many gaps in scientific knowledge about the clinical significance of pharmacokinetic natural product–drug interactions (NPDIs) in which the natural product (NP) is the precipitant and a conventional drug is the object. The National Center for Complimentary and Integrative Health created the Center of Excellence for NPDI Research (NaPDI Center) (www.napdi.org) to provide leadership and guidance on the study of pharmacokinetic NPDIs. A key contribution of the Center is the first user-friendly online repository that stores and links pharmacokinetic NPDI data across chemical characterization, metabolomics analyses, and pharmacokinetic in vitro and clinical experiments (repo.napdi.org). The design is expected to help researchers more easily arrive at a complete understanding of pharmacokinetic NPDI research on a particular NP. The repository will also facilitate multidisciplinary collaborations, as the repository links all of the experimental data for a given NP across the study types. The current work describes the design of the repository, standard operating procedures used to enter data, and pharmacokinetic NPDI data that have been entered to date. To illustrate the usefulness of the NaPDI Center repository, more details on two high-priority NPs, cannabis and kratom, are provided as case studies.

**SIGNIFICANCE STATEMENT:**

The data and knowledge resulting from natural product–drug interaction (NPDI) studies is distributed across a variety of information sources, rendering difficulties to find, access, and reuse. The Center of Excellence for NPDI Research addressed these difficulties by developing the first user-friendly online repository that stores data from in vitro and clinical pharmacokinetic NPDI experiments and links them with study data from chemical characterization and metabolomics analyses of natural products that are also stored in the repository.

## Introduction

Natural products (NPs) include herbal and other botanical products (Paine and Roe, 2018). Pharmacokinetic interactions involving NPs and conventional [e.g., approved by the US Food and Drug Administration (FDA)] drugs could result in reduced treatment efficacy or adverse effects (Paine et al., 2018). Although up to 88% of older adults use herbal medicinal products concurrently with conventional drugs (Batanero-Hernán et al., 2017), there are many gaps in scientific knowledge about the clinical significance of pharmacokinetic NP–drug interactions (NPDIs) in which the NP is the precipitant and a conventional drug is the object. Although 6 of the 40 top-selling herbal medicinal products in 2017 were implicated in clinically significant pharmacokinetic NPDIs, there was minimal or no supporting clinical evidence for potential NPDIs involving nine products (Spanakis et al., 2019). Similarly, data were insufficient to conclude the clinical relevance of 11 of the 15 potential pharmacokinetic NPDIs involving antiretroviral drugs (Fasinu et al., 2015).

There are several unique challenges associated with pharmacokinetic NPDI research, including the large variability of phytoconstituents among marketed products, difficulty extrapolating results from animal and/or in vitro models to humans, variability in study design, and inadequate methods (Paine et al., 2018). Based on these knowledge gaps and challenges, the National Center for Complimentary and Integrative Health created the Center of Excellence for NPDI Research (NaPDI Center; www.napdi.org) to provide leadership and guidance on the study of pharmacokinetic NPDIs (Paine et al., 2018).

One objective of the NaPDI Center is to develop and apply a set of Recommended Approaches to determine the clinical relevance of pharmacokinetic NPDIs (Johnson et al., 2018; Paine et al., 2018; Kellogg et al., 2019). A key deliverable of the Center is the development of an online repository for data generated by the NaPDI Center (repo.napdi.org). The repository combines data currently distributed across a variety of information sources into a single user-friendly format complemented by an information portal. This portal, also developed by the NaPDI Center, disseminates the Recommended Approaches (Johnson et al., 2018; Paine et al., 2018; Kellogg et al., 2019) on the optimal conduct of pharmacokinetic NPDI studies (napdicenter.org). Combined, these new resources will help advance pharmacokinetic NPDI research by providing Recommended Approaches and novel pharmacokinetic NPDI data.

Pharmacokinetic NPDI data include chemical characterization of NPs, metabolomics analyses, and in vitro and clinical pharmacokinetic experimental results. This new repository stores data from all of these types of investigations. It provides a user-friendly interface that enables users with limited informatics skills to effectively explore relevant data (Li, 2015). As of March 2020, coverage of the repository is limited to four carefully selected high-priority NPs based on a systematic method for the purpose of demonstrating the Recommended Approaches (Johnson et al., 2018): cannabis (*Cannabis sativa*), goldenseal (*Hydrastis canadensis*), green tea (*Camellia sinensis*), and kratom (*Mitragyna speciosa*). A prior Recommended Approach (Johnson et al., 2018) reported the inclusion of licorice (*Glycyrrhiza* spp.). The Center later replaced licorice with kratom to 1) keep pace with public health needs in the face of an ever-changing NP market (Gaston et al., 2020) and 2) omit redundancy with the research efforts of a longstanding botanical center (https://pcrps.pharmacy.uic.edu/our-centers/uic-nih-center-for-botanical-dietary-supplements-research/).

The current work describes the design of the repository, standard operating procedures (SOPs) used to enter data, and pharmacokinetic NPDI data that have been entered to date. To illustrate the usefulness of the NaPDI Center repository, more details on two high-priority NPs, cannabis and kratom, are provided as case studies.

## Materials and Methods

### Construction and Content

#### Studies Conducted by NaPDI Center Investigators.

**Fig. 1.**
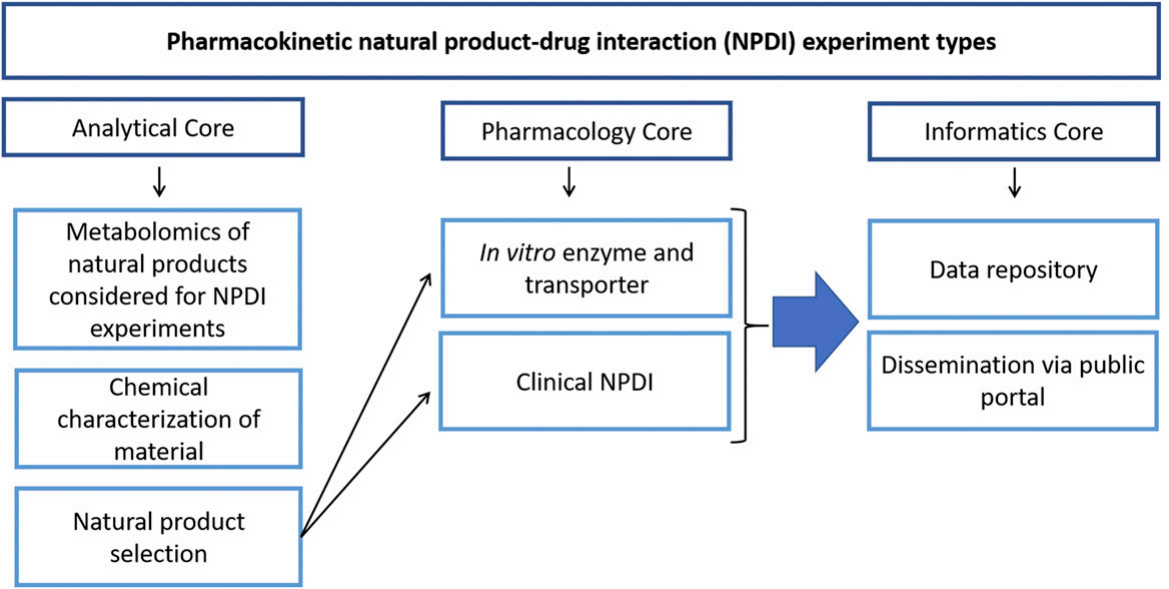
Types of pharmacokinetic NPDI experiments conducted by the NaPDI Center.

To date, the repository has focused on original pharmacokinetic NPDI research conducted by NaPDI Center investigators, who are organized into three cores with complementary expertise (Fig. 1).

The Analytical Core is composed of NP chemists, analytical chemists, and clinical pharmacologists and serves multiple functions. This core chemically characterizes multiple commercially available products of a given NP, determines the contents of constituents in these products, and provides guidance on the proper selection of one or more commercially available products to be tested by the Pharmacology Core. The core also analyzes plasma and urine samples obtained from pharmacokinetic clinical studies for NP constituents and object drugs.

**Fig. 2.**
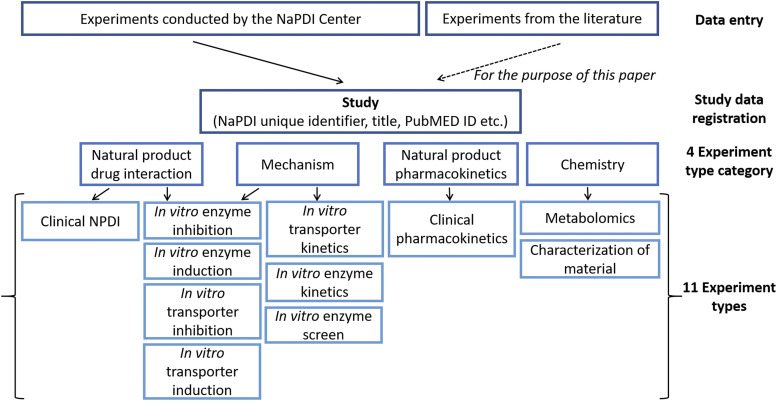
Data resulting from experiments conducted by the NaPDI Center and from experiments reported in the literature are entered into the repository. Each “Study” record describes an activity that resulted in data from one or more related experiments. Each experiment record is assigned 1 of the 11 experiment types offered that provides the appropriate format for recording experimental conditions and results following instructions provided in the 11 SOPs.

The Pharmacology Core is composed of clinical pharmacologists and medicinal chemists. This core designs and conducts rigorous experiments to evaluate the potential for NPs to precipitate pharmacokinetic interactions with certain object drugs. The core also characterizes the pharmacokinetics of select NP constituents in human subjects. The data obtained are used to develop physiologically based pharmacokinetic models that can be applied to other object drugs and patient populations of interest. Figure 2 shows the variety of different experiment types that the repository supports to store data from the NaPDI Center’s interaction projects.

The Informatics Core (Fig. 1) is composed of biomedical informaticists, computer scientists, and communication experts. This core compiles all data generated from NaPDI Center research activities into the data repository, which is accessible via the information portal. Prior to public release, NaPDI Center data are only accessible to researchers approved to access the site. Contributing researchers indicate when to make the data public. The data are made available according to a Recommended Approach for making pharmacokinetic NPDI research data findable, accessible, interoperable, and reusable (FAIR; https://www.w3id.org/hclscg/npdi).

#### Data Types.

A variety of data types are produced from pharmacokinetic NPDI studies (Supplemental Table 1). Initially, the specification and subsequent characterization of the NP source materials generated a diverse set of data, including chromatograms from conventional high-pressure liquid chromatography with UV detection and ultrahigh-pressure liquid chromatography–mass spectrometry methods, spectral data from nuclear magnetic resonance and circular dichroism, and bioactivity fractionation data. These data include instrument tracings that are often not retrievable in digitized form. Hence, the scanned image files are archived in the repository. Quantitative data on NP source materials, such as content of individual phytoconstituents and specific impurities or contaminants, are organized in tabular format.

The types of data generated from in vitro NPDI studies vary across the range of human-derived in vitro test systems, including enzymatic reactions involving recombinant enzymes, human tissue fractions (e.g., human liver microsomes), or cultured cells (e.g., hepatocytes), and drug transport experiments measuring uptake into membrane vesicles or efflux from transfected cells. Currently, the data repository tracks 82 measurements for quantitative data resulting from NPDI experiments. The full list is provided in Supplemental Table 1. Included in the list are, for example, percent inhibition, IC_50_, K_m_, and V_max_.

In addition, data generated from inhibition experiments involving drug metabolizing enzymes or transporters differ from those generated from induction experiments. Thus, the repository provides separate sets of data fields for each of these in vitro systems and mechanisms (Supplemental Table 1).

Pharmacokinetic data generated from clinical NPDI studies include human subject demographics, concentration-time data, and key pharmacokinetic endpoints (e.g., oral clearance, renal clearance, apparent volume of distribution, half-life, area under the plasma-concentration vs. time curve, maximum plasma concentration, and time to reach maximum concentration). Statistical analyses of primary and secondary pharmacokinetic endpoints generated additional data sets.

#### Data Findability, Accessibility, Interoperability, and Reusability.

There is a growing recognition by both researchers and funding agencies that pharmacokinetic NPDI study data sets should be more FAIR (National Center for Complementary and Integrative Health, 2019). The NaPDI Center repository is designed to ensure that data satisfy these four foundational principles of good data management and stewardship. Table 1 summarizes the specific features of the repository that support FAIR pharmacokinetic NPDI data. Each feature is described in greater detail in a public and participative report that the NaPDI Center is developing in collaboration with the World Wide Web Consortium Semantic Web in Health Care and Life Sciences Community Group (https://www.w3id.org/hclscg/npdi).

**TABLE 1:** Data in the NaPDI Center repository are FAIR

FAIR	General
Findability	Each data set receives a unique identifier.
	Study and experiment metadata are published using a machine-readable format. The update frequency of the data is available for each study and experiment.
Accessibility	Full data sets are downloadable.
	Data are accessible in a variety of formats and can be retrieved using a REST-full API.
	The repository uses HTTP content negotiation to serve data requests.
	The repository search capabilities support simple search and advanced faceted search.
Interoperability	Data sets use data elements from existing ontologies and terminologies as much as possible.
	NMR and MS results are reported following accepted standards.
Reusability	Standard operating procedures are publicly available.
	Experiments are described in clear detail.
	Study and experiment metadata provide clear licensing requirements.
	Repository users can provide feedback and ask questions.
	Raw spectral data are available using an open file format.

API, application programming interface; HTTP, hypertext transfer protocol; MS, mass spectrometry; NMR, nuclear magnetic resonance; REST, representational state transfer.

#### Standard Operating Procedures for Data Entry.

A major feature of the repository is that data are entered using validated SOPs. There are currently 11 SOPs, one for each experiment type listed in Figure 2. Data collection forms have been developed for both internal and external NPDI researchers, such as contract research organizations. These forms are based closely on the SOP documents. Both the SOPs and data entry forms are publicly available on GitHub (https://github.com/dbmi-pitt/NaPDI-SOPs), and the SOP document for enzyme inhibition experiment type is provided as an example in Supplemental Data (Boyce et al., 2020).

#### Quality Control and Validation Processes.

Given the variety of data types, close attention must be paid to enable accurate tracking and meticulous organization of the generated data. The structure, data organization, and concepts effectively used by the University of Washington’s Drug Interaction Database (Hachad et al., 2010), now Drug Interaction Solutions (www.druginteractionsolutions.org), have been applied to the NaPDI Center repository. These features have been validated over time with feedback from a large user base. To ensure the quality and consistency of the entry process, data are entered by experienced curators who are well versed in drug interactions using the aforementioned SOPs. All data entry undergoes review by a second reviewer prior to public release.

#### Current Status of the Repository.

An overview of data entered into the NaPDI Center repository is provided for two of the high-priority NPs selected as case studies: cannabis (*C. sativa*) and kratom (*M. speciosa*). These NPs were chosen due to increasing use and public interest. Neither NP has been well studied with respect to NPDI potential. In the United States, a majority of states have legalized marijuana for recreational and/or medical purposes. Moreover, a growing number of products containing the nonpsychotropic phytocannabinoid cannabidiol are marketed every year. These products include the FDA-approved drug Epidiolex and numerous unapproved tinctures, oils, and extracts. Kratom, a member of the coffee family native to Southeast Asia, is touted for its analgesic and stimulant effects. Warnings about kratom toxicity have been raised by the US FDA and the Centers for Disease Control and Prevention (Food and Drug Administration, 2019; Gershman, 2019). Calls to US poison centers involving kratom exposures from 2011 to 2017 increased 52-fold, from 13 to 682, with more than one-third of the calls reported involving co-consumption with prescription or illicit drugs (Post et al., 2019).

Each case study begins with a summary of NaPDI Center research activities focusing on each NP as a precipitant of pharmacokinetic NPDIs. A description follows about how published evidence was added to the repository to both complement the data generated by the NaPDI Center and provide researchers with a more complete picture of the pharmacokinetic interaction potential for each NP.

#### NPDI Study Process.

**Fig. 3.**
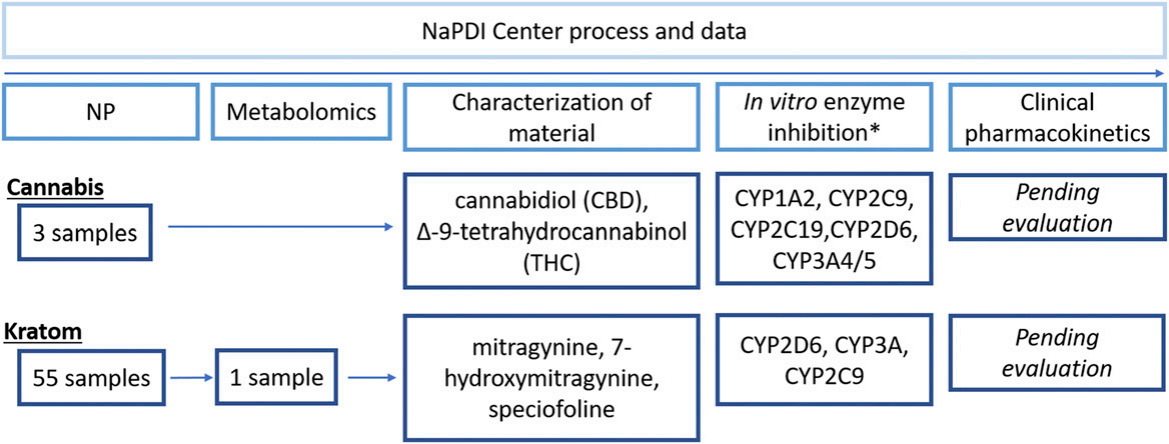
Process and data undertaken by the NaPDI Center for the study of pharmacokinetic NPDIs precipitated by cannabis and kratom. *The Analytical Core did not source or characterize the cannabis study materials, whereas it conducted both investigations for kratom. The purified cannabis study materials were purchased from a commercial vendor.

Four steps are crucial for conducting a rigorous research study on a given pharmacokinetic NPDI: NP selection; sourcing and chemical characterization of different commercial products of the selected NP; in vitro assessment of inhibition or induction of drug metabolizing enzymes and transporters by the NP; and, if necessary based on the prior data, a clinical study of potential pharmacokinetic NPDIs in human subjects (Fig. 3).

The upper half of Figure 3 shows the cannabis studies conducted by the NaPDI Center as of March 2020. Chemical characterization data for two products were obtained from the National Center for Natural Products Research at the University of Mississippi. One product was an extract enriched in delta-9-tetrahydrocannabinol (THC) and the other was an extract enriched in cannabidiol (CBD). Purified THC and CBD were tested as inhibitors of five major cytochrome P450 (P450) enzymes, namely, CYP1A2, CYP2C9, CYP2C19, CYP2D6, and CYP3A4/5. Results informed the design of an ongoing clinical cannabis-drug interaction study.

The lower half of Figure 3 shows the kratom studies conducted by the NaPDI Center as of March 2020. The Analytical Core conducted a metabolomics study involving 55 kratom products, informing the selection of one product for further in vitro and clinical studies. The selection criteria followed a published NaPDI Center Recommended Approach (Kellogg et al., 2019). The Analytical Core conducted chemical characterization of the selected product to quantify mitragynine, 7-hydroxymitragynine, and speciofoline (Fig. 3). Extracts prepared from three kratom products, including one that was eventually selected for the clinical study, were tested by the Pharmacology Core as inhibitors of three major P450s, specifically CYP2C9, CYP2D6, and CYP3A4/5. As with cannabis, the in vitro results informed the design of the ongoing clinical kratom-drug interaction study.

#### Literature Search Process.

Additional data were identified from peer-reviewed published reports in order for the data repository to provide greater research context for the NaPDI Center–conducted studies. Systematic literature searches were designed to retrieve studies on NP constituent pharmacokinetics and drug interactions involving either cannabis or kratom. The final search strategies are available in the Appendix. Queries were run in PubMed in July 2018 and again in February 2020.

The screening of titles and abstracts, and subsequently full text articles, was completed independently and in duplicate to identify experiments of the types shown in Figure 2. Mechanistic experiments of interest included assessing the NP as an inhibitor or inducer of P450s, UDP-glucuronosyltransferases (UGTs), and transporters. Clinical experiments of interest included pharmacokinetic NPDIs involving cannabis or kratom. Experiments involving only synthetic analogs, pharmacodynamics, or nonhuman animal studies and review articles were excluded. Full text articles available only in non-English languages were also excluded. Published reports cited in a recent review by the NaPDI Center (Cox et al., 2019) on cannabis pharmacology and pharmacokinetics (*n* = 6) were added to the screening results.

#### Data Entry of Published Literature and Pharmacokinetic NPDI Studies.

Data from the included published reports were entered into the repository following the aforementioned SOPs (Boyce et al., 2020). When available, exact values from the text were entered. Otherwise, estimates were made from the study figures. Data extracted from each report were marked as “draft” during initial data entry and “pending” upon completion of data entry. After quality assurance by a second reviewer, the extracted data were made public. Data entry issues were tracked and addressed until quality assurance was complete for all studies.

## Results

### Construction and Content

As of April 2020, the NaPDI Center repository contains data from 777 experiments (Table 2). Currently, the most common experiment types are in vitro enzyme inhibition (405), in vitro enzyme induction (99), in vitro transport inhibition (78), and clinical pharmacokinetic NPDIs (57). The remaining 138 experiments are of various other types supported by the repository. In line with FAIR recommendations, every experiment is assigned a unique and persistent identifier that also resolves to a downloadable copy of a data set. A clear description of each experiment’s conditions is provided by the repository website. The repository publishes metadata about each experiment that is machine readable and confirmed to work with Google’s Dataset Search (https://datasetsearch.research.google.com/). To provide the most optimal experience to the researcher or editor wanting to search for data in the repository, an interactive and silent guided tour is provided on the home page (see the screen capture video in Supplemental Data).

**TABLE 2:** Number of experiments deposited in the NaPDI Center data repository as of April 2020 detailed for cannabis (*C. sativa*) and kratom (*M. speciosa*)

NaPDI Center repository (as of April 2020)	All high-priority NPs	Cannabis	Kratom
Chemical characterization experiments			
Characterization of NP study material	9	3	1
Metabolomics	3	0	1
In vitro experiments	99	5	61
Enzyme induction			
Enzyme inhibition	405	116	99
Enzyme kinetics	16	9	3
Enzyme screen	1	0	1
Transporter induction	55	13	32
Transporter inhibition	78	25	4
Transporter kinetics	34	2	10
Clinical NPDI experiments	57	33	0
Pharmacokinetic NPDI			
NP pharmacokinetics	20	7	0
Total	777	213	212

### Utility

This section reports the results of NaPDI Center repository data entry of the two high-priority NPs selected as case studies: cannabis (*C. sativa*) and kratom (*M. speciosa*).

#### Cannabinoids.

**Fig. 4.**
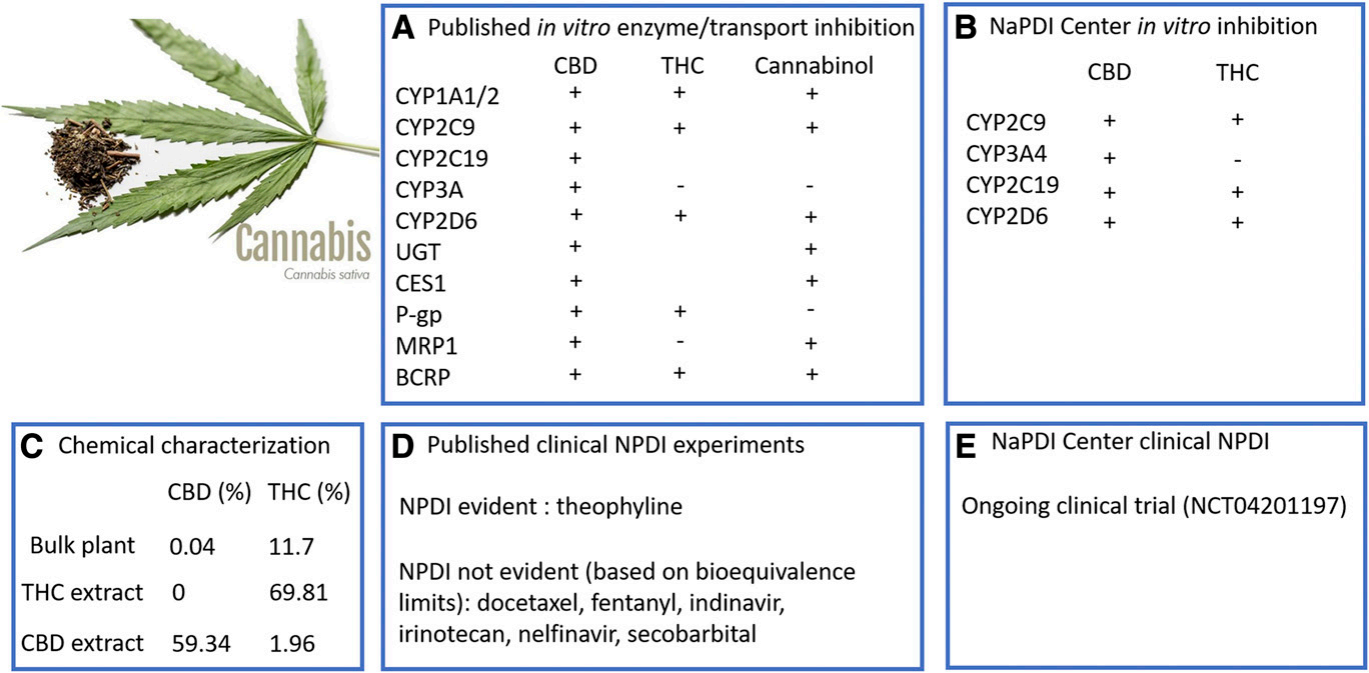
Overview of reported NPDI data for cannabis from both NaPDI Center studies and peer-reviewed publications.

Figure 4 provides an overview of reported NPDI data for cannabis from both NaPDI Center studies and peer-reviewed published reports. Links to the specific experiments are provided in Supplemental Table 2.

Chemical characterization data obtained from the National Center for Natural Products Research (https://pharmacy.olemiss.edu/ncnpr/) for two cannabis extracts and bulk plant material provided the exact concentration of CBD, THC, and other cannabinoids. The data confirmed the CBD-enriched extract (CBD 59.34%, THC 1.96%) to have a higher concentration of CBD than the bulk plant (CBD 0.04%, THC 11.7%) or THC-enriched extract (CBD 0%, THC 69.81%) (Fig. 4). NaPDI Center experiments confirmed that CBD inhibited CYP2C9, CYP3A4/5, CYP2C19, and CYP2D6 and that THC inhibited CYP2C9, CYP2C19, and CYP2D6 (unpublished data).

Data from a total of 22 published in vitro reports focusing on cannabis-drug interactions were entered into the repository (Holland et al., 2006, 2007, 2008; Zhu et al., 2006; Watanabe et al., 2007; Mazur et al., 2009; Alhamoruni et al., 2010; Tournier et al., 2010; Yamaori et al., 2010, 2011a,b, 2012, 2013, 2014, 2015; Jiang et al., 2011, 2013; Arnold et al., 2012; Al Saabi et al., 2013; Feinshtein et al., 2013a,b; Qian et al., 2019). As Figure 4 shows, experiments using either human liver microsomes or recombinant baculovirus–transfected insect cells expressing specific P450/UGT isoforms reported that cannabinoids inhibit CYP1A1, CYP1A2, CYP2C9, CYP2C19, CYP2D6, CYP3A4/5, and UGT (Mazur et al., 2009; Yamaori et al., 2010, 2011a,b, 2012, 2013; Al Saabi et al., 2013; Jiang et al., 2013; Qian et al., 2019). Yamaori et al. reported that CBD mechanistically inhibited CYP1A1 in vitro in recombinant baculovirus transfected insect cells. Qian et al. reported that CBD and cannabinol inhibited carboxylesterase 1 in vitro in human embryonic kidney 293 cells (Qian et al., 2019).

In vitro inhibition of P-glycoprotein–mediated efflux transport was reported for THC from experiments using transfected human embryonic kidney cells and for CBD using BeWo choriocarcinoma, LLC-PK1/MDR1, or MCF7/P-gp cells (Zhu et al., 2006; Tournier et al., 2010; Feinshtein et al., 2013a). An experiment using a human ovarian carcinoma cell line reported that cannabinol inhibited the efflux transporter multidrug resistance-associated protein 1 (MRP1 or ABCC1) (Holland et al., 2008). Experiments using BeWo, Jar, MCF7/P-gp, and MEF3.8/Bcrp A2 cell lines reported that CBD inhibited breast cancer resistance protein (BCRP or ABCG2), an effect that was reported for THC and cannabinol using the cell line MEF3.8/Bcrp A2 (Holland et al., 2008; Feinshtein et al., 2013b).

A total of nine published clinical reports focusing on pharmacokinetic cannabis-drug interactions were entered into the repository (Dalton et al., 1976; Jusko et al., 1978; Perez-Reyes et al., 1988; Kosel et al., 2002; Haney et al., 2003; Engels et al., 2007; Kleinloog et al., 2012; Stott et al., 2013; Manini et al., 2015). Only one study reported an interaction involving smoked *C. sativa*, which was observed to increase the clearance of the CYP1A2 substrate theophylline (Jusko et al., 1978). Clinical pharmacokinetic interactions between cannabis and docetaxel, fentanyl, indinavir, irinotecan, nelfinavir, or secobarbital were not evident based on bioequivalence limits (Dalton et al., 1976; Kosel et al., 2002; Engels et al., 2007; Manini et al., 2015). One clinical study compared the plasma concentrations of THC and CBD under fasting and fed conditions (Stott et al., 2013), whereas another study reported estimated pharmacokinetic parameters for THC (Kleinloog et al., 2012).

#### Kratom.

**Fig. 5.**
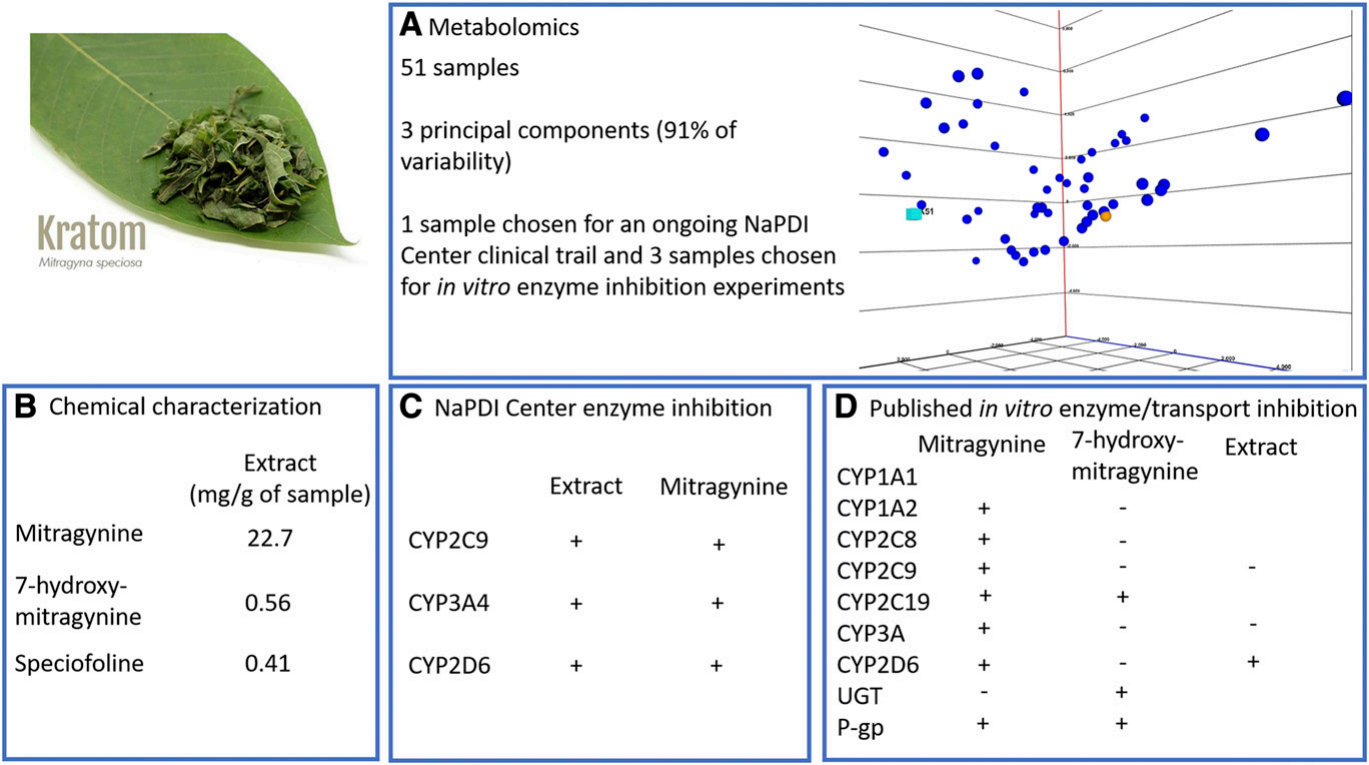
Overview of reported NPDI data for kratom from both NaPDI Center studies and peer-reviewed publications. The results shown in boxes “b” and “c” are for the product chosen from the metabolomics study (light blue highlight in box “a”).

Figure 5 provides an overview of pharmacokinetic NPDI data for kratom from both NaPDI Center studies and peer-reviewed published reports. Links to the specific experiments are provided in Supplemental Table 3.

The Analytical Core’s metabolomics analysis of 51 kratom products highlighted differences in chemical compound profiles depending on the manufacturer, form, and geographic location where the plants grew. A principal components analysis of the data identified three principal components explaining 91% of the variability across the features included in the metabolomics analysis.

Chemical characterization of the methanolic kratom extract used in the ongoing NaPDI in vitro and clinical studies (made from a clinical product) identified mitragynine (22.7 mg/g of sample), 7-hydroxymitragynine (0.57 mg/g of sample), and speciofoline (0.41 mg/g of sample). The in vitro inhibition studies showed that both the methanolic kratom extract and mitragynine inhibited CYP2C9, CYP2D6, and CYP3A4/5 by differing extents (unpublished observations).

Data from nine published in vitro studies were entered into the repository (Hanapi et al., 2010, 2013; Kong et al., 2011; Haron and Ismail, 2014; Manda et al., 2014; Meyer et al., 2015; Kamble et al., 2019, 2020; Rusli et al., 2019). One study using recombinant P450 enzymes reported that a methanolic extract of kratom inhibited CYP2D6 but not CYP2C9 or CYP3A4 (Hanapi et al., 2010). One study using pooled human liver microsomes reported inhibition of CYP2C19 by 7-hydroxymitragynine (Kamble et al., 2020), whereas another study using recombinant enzymes reported inhibition of UGT1A1 by 7-hydroxymitragynine (Haron and Ismail, 2014).

Mitragynine inhibition of CYP2D6 was reported in three different studies using pooled human liver microsomes (Kamble et al., 2020), recombinant P450s (Hanapi et al., 2013), and a high-throughput in vitro fluorescent P450 assay (Kong et al., 2011). Mitragynine inhibition of CYP3A and CYP2C19 was reported with pooled human liver microsomes (Kamble et al., 2020) and the in vitro fluorescent P450 assay (Kong et al., 2011). Mitragynine inhibition of CYP2C8 was reported with pooled human liver microsomes (Kamble et al., 2020), CYP1A2 with an in vitro fluorescent P450 assay (Kong et al., 2011), and CYP2C9 with recombinant P450 enzymes (Hanapi et al., 2013).

Three studies reported inhibition of P-glycoprotein by mitragynine, two using Caco-2 cells (Meyer et al., 2015; Rusli et al., 2019), and one using MDCK-transfected cells (Manda et al., 2014). The same MDCK-transfected cell study reported inhibition of P-glycoprotein by 7-hydroxymitragynine. One study reported CYP3A4 as the primary metabolizing enzyme for mitragynine (Kamble et al., 2019). Another study reported downregulation of P-glycoprotein in Caco-2 cells by mitragynine (Rusli et al., 2019).

## Discussion

Although rigorous pharmacokinetic NPDI research can mitigate adverse interactions, the data and knowledge resulting from these experiments are currently distributed across a variety of information sources, making them difficult to find, access, and reuse. The new NaPDI Center repository is the first user-friendly online repository that stores and links pharmacokinetic NPDI data across chemical characterization, metabolomics analyses, and pharmacokinetic in vitro and clinical experiments. The design is expected to help researchers more easily arrive at a complete understanding of pharmacokinetic NPDI research on a particular NP. The repository will also facilitate multidisciplinary collaborations, as the repository links all of the experimental data for a given NP across the study types. For example, the repository links chemical characterization data with data from in vitro and clinical experiments and vice versa. This feature should help facilitate communication between multidisciplinary researchers working on different aspects of a particular pharmacokinetic NPDI.

The mission of the NaPDI Center is to provide leadership and guidance on the study of pharmacokinetic NPDIs. Currently, only data on the four high-priority NPs under study by the NaPDI Center have been entered in the repository. Future work hopes to expand the repository to include a larger selection of NPs and engage NPDI researchers external to the NaPDI Center. Toward that goal, pilot work is completed that includes data from experiments involving P450 inhibition by three licorice species (i.e., *Glycyrrhiza glabra*, *G. uralensis*, and *G. inflata*) (Li et al., 2017). The published report includes pharmacokinetic NPDI data specific to extracts of each licorice species and for individual constituents present in some or all licorice species. The repository links all of these data in a manner that allows researchers to explore P450 inhibition by licorice from a variety of perspectives (i.e., single or multiple licorice species and single or multiple licorice constituents). It is useful to emphasize that the NaPDI Center repository currently focuses on pharmacokinetic NPDI data. At the present time there are no plans to integrate pharmacodynamic NPDI data. Though it has not been the focus to date, the format for data in the NaPDI data repository allows for setting the NP as the object drug, and there are a handful of experiments in the repository of this kind that have been entered as test cases. The inclusion of this kind of data might become the focus in the future depending on feedback from the NPDI research community and other stakeholders.

Building upon this strong foundation, the NaPDI Center plans to create novel information visualizations to provide researchers a complete evidence-based overview of the potential of each NP to precipitate pharmacokinetic NPDIs. The Center also plans to permit other researchers to submit data using files or the repository’s web-based application programming interface with the goal of supporting medium- to high-throughput assays that generate megabytes or gigabytes of data. Researchers external to the NaPDI Center can enter data by first requesting an account and then following the SOP documents during data entry. After a researcher’s data entry is completed, a trained individual within the NaPDI Center will inspect the entered data before public release.

Finally, the NaPDI Center plans to implement automatic FAIR quality analytic reports that will run each time a data submitter marks a new study entry as “pending.” Issues identified from the reports can then be addressed promptly by the data submitter. These functionalities, combined with the existing functionalities of the NaPDI Center repository, seek to facilitate pharmacokinetic NPDI research with the long-range goal of mitigating adverse interactions and improving public health.

## Abbreviations

CBD: cannabidiol
FAIR: findable, accessible, interoperable, and reusable
FDA: US Food and Drug Administration
NaPDI Center: Center of Excellence for Natural Product–Drug Interaction Research
NP: natural product
NPDI: NP-drug interaction
P450: cytochrome P450
SOP: standard operating procedure
THC: tetrahydrocannabinol
UGT: UDP-glucuronosyltransferase

## Appendix: Search Strategy for Cannabis

### Clinical Studies

(Clinical Trial [PT] AND (Cannabis[MeSH Terms] OR “cannabinoids”[All Fields] OR “Cannabidiol”[All Fields] OR “CBD”[All Fields] OR “Cannabinol”[All Fields] OR “Dronabinol”[All Fields] OR “delta(9)-THC”[All Fields] OR “9-ene-Tetrahydrocannabinol”[All Fields] OR “9 ene Tetrahydrocannabinol”[All Fields] OR “THC”[All Fields] OR “delta(1)-Tetrahydrocannabinol”[All Fields] OR “delta(1)-THC”[All Fields] OR “delta(9)-Tetrahydrocannabinol”[All Fields] OR “Tetrahydrocannabinol”[All Fields] OR “Tetrahydrocannabinol, (6a-trans)-Isomer”[All Fields] OR “Tetrahydrocannabinol, Trans-Isomer”[All Fields] OR “Tetrahydrocannabinol, Trans Isomer”[All Fields] OR “Tetrahydrocannabinol, (6aS-cis)-Isomer”[All Fields] OR “Tetrahydrocannabinol, Trans-(+-)-Isomer”[All Fields] OR “Marinol”[All Fields] OR “Tetrahydrocannabinol, (6aR-cis)-Isomer”[All Fields] OR “delta-9-tetrahydrocannabinol”[All Fields] OR “(-)delta-9-tetrahydrocannabinol”[All Fields] OR “THC”[All Fields]) AND “drug interactions”[All Fields]) NOT Review [PT].

Mechanistic NPDI studies useful for inferring NPDIs:

Step 1) Log into My NCBI and go to Pubmed: https://www.ncbi.nlm.nih.gov/pubmed/.

Step 2) In the advanced search form, clear the search history.

Step 3) Paste in this query into builder (using “edit”) and click “add to history”—this step is referred to as “#1” in the rest of this search strategy:

“Cytochrome P-450 Enzyme System”[MeSH Terms] OR “Cytochrome P450 Family 1”[MeSH Terms] OR “Cytochrome P450 Family 2”[MeSH Terms] OR “Cytochrome P450 Family 3”[MeSH Terms] OR CYP1A1[All Fields] OR CYP1A2[All Fields] OR CYP1A3[All Fields] OR CYP1A4[All Fields] OR CYP1A5[All Fields] OR CYP2D6[All Fields] OR CYP2C9[All Fields] OR CYP2A6[All Fields] OR CYP2C8[All Fields] OR CYP2C19[All Fields] OR CYP2B6[All Fields] OR CYP2B1[All Fields] OR CYP2E1[All Fields] OR CYP3A4[All Fields] OR CYP3A5[All Fields] OR UGT1[All Fields] OR UGT1A1[All Fields] OR UGT1A3[All Fields] OR UGT1A4[All Fields] OR UGT1A5[All Fields] OR UGT1A6[All Fields] OR UGT1A7[All Fields] OR UGT1A8[All Fields] OR UGT1A9[All Fields] OR UGT1A10[All Fields] OR UGT2[All Fields] OR UGT2A1[All Fields] OR UGT2A2[All Fields] OR UGT2A3[All Fields] OR UGT2B4[All Fields] OR UGT2B7[All Fields] OR UGT2B10[All Fields] OR UGT2B11[All Fields] OR UGT2B15[All Fields] OR UGT2B17[All Fields] OR UGT2B28[All Fields] OR B3GAT1[All Fields] OR B3GAT2[All Fields] OR B3GAT3[All Fields].

Step 4) Paste in this query into builder (using “edit”) and click “add to history”—this is referred to as “#2” in the rest of this search strategy.

“Solute Carrier Proteins”[MeSH Terms] OR “Membrane Transport Proteins”[MeSH Terms] OR “P-gp”[All Fields] OR “p-glycoprotein”[All Fields] OR BCRP[All Fields] OR OCT2[All Fields] OR “Organic Cation Transporter 2”[MeSH Terms] OR “Organic Cation Transport Proteins”[MeSH Terms] OR MATE1[All Fields] OR “SLC4A Proteins”[MeSH Terms] OR MATE-2K[All Fields] OR “SLC4A Proteins”[MeSH Terms] OR OATP[All Fields] OR OAT1[All Fields] OR “Organic Anion Transport Protein 1”[MeSH Terms] OR OAT3[All Fields] OR UGT1[All Fields] OR “Glucuronosyltransferase”[MeSH Terms] OR ABC[All Fields] OR “ATP-Binding Cassette Transporters”[MeSH Terms]

Step 5) Paste in this query into builder (using “edit”) and click “add to history”—this step is referred to as “#3” in the rest of this search strategy.

(Cannabis[MeSH Terms] OR “cannabinoids”[All Fields] OR “Cannabidiol”[All Fields] OR “CBD”[All Fields] OR “Cannabinol”[All Fields] OR “Dronabinol”[All Fields] OR “delta(9)-THC”[All Fields] OR “9-ene-Tetrahydrocannabinol”[All Fields] OR “9 ene Tetrahydrocannabinol”[All Fields] OR “THC”[All Fields] OR “delta(1)-Tetrahydrocannabinol”[All Fields] OR “delta(1)-THC”[All Fields] OR “delta(9)-Tetrahydrocannabinol”[All Fields] OR “Tetrahydrocannabinol”[All Fields] OR “Tetrahydrocannabinol, (6a-trans)-Isomer”[All Fields] OR “Tetrahydrocannabinol, Trans-Isomer”[All Fields] OR “Tetrahydrocannabinol, Trans Isomer”[All Fields] OR “Tetrahydrocannabinol, (6aS-cis)-Isomer”[All Fields] OR “Tetrahydrocannabinol, Trans-(+-)-Isomer”[All Fields] OR “Marinol”[All Fields] OR “Tetrahydrocannabinol, (6aR-cis)-Isomer”[All Fields] OR “delta-9-tetrahydrocannabinol”[All Fields] OR “(-)delta-9-tetrahydrocannabinol”[All Fields] OR “THC”[All Fields]).

Step 6) Paste in this query into builder (using “edit”) and click “add to history”—this is referred to as “#4” in the rest of this search strategy.

(Pharmacokinetics[MeSH Terms] OR pharmacokinetic[All Fields]) or (inhibit[All Fields] or inhibition[All Fields]) OR substrate[All Fields]

Step 7) Paste in this query into builder (using “edit”) and click “add to history”—this step is referred to as “#5” in the rest of this search strategy.

#3 AND #4 AND (#1 OR #2) AND “humans”[MeSH Terms].

## Search Strategy for Kratom

### Clinical Studies

(Clinical Trial [PT] AND (“mitragynine”[All Fields] OR “mitragynine ethanedisulfonate”[All Fields] OR “SK and F 12711”[All Fields] OR “SKF 12711”[All Fields] OR “SK and F-12711”[All Fields] OR “mitragynine, (16E)-isomer”[All Fields] OR “mitragynine, (3beta,16E)-isomer”[All Fields] OR “mmitragynine, (3beta,16E,20beta)-isomer”[All Fields] OR “kratom alkaloids”[All Fields] OR “kmitragynine monohydrochloride”[All Fields] OR “Mitragyna speciosa”[All Fields] OR “Nauclea speciose”[All Fields] OR “Biak-biak”[All Fields] OR “Cratom”[All Fields] OR “Gratom”[All Fields] OR “Ithang”[All Fields] OR “Kakuam”[All Fields] OR “Katawn”[All Fields] OR “Kedemba”[All Fields] OR “Ketum”[All Fields] OR “Krathom”[All Fields] OR “Kraton”[All Fields] OR “Kratum”[All Fields] OR “Madat”[All Fields] OR “Mambog”[All Fields] OR “Mitragynine”[All Fields] OR “Mitragynine extract”[All Fields] OR “Thang”[All Fields] OR “Thom”[All Fields] OR “7-hydroxymitragynine”[All Fields] OR “7-hydroxy-mitragynine”[All Fields] OR “mitragynine pseudoindoxyl”[All Fields] OR “Paynantheine”[All Fields]) AND “drug interactions”[All Fields]) NOT Review [PT]

Mechanistic NPDI studies useful for inferring NPDIs:

Step 1) Log into My NCBI and go to Pubmed: https://www.ncbi.nlm.nih.gov/pubmed/.

Step 2) In the advanced search form, clear the search history.

Step 3) Paste in this query into builder (using “edit”) and click “add to history”—this is referred to as “#1” in the rest of this search strategy:

“Cytochrome P-450 Enzyme System”[MeSH Terms] OR “Cytochrome P450 Family 1”[MeSH Terms] OR “Cytochrome P450 Family 2”[MeSH Terms] OR “Cytochrome P450 Family 3”[MeSH Terms] OR CYP1A1[All Fields] OR CYP1A2[All Fields] OR CYP1A3[All Fields] OR CYP1A4[All Fields] OR CYP1A5[All Fields] OR CYP2D6[All Fields] OR CYP2C9[All Fields] OR CYP2A6[All Fields] OR CYP2C8[All Fields] OR CYP2C19[All Fields] OR CYP2B6[All Fields] OR CYP2B1[All Fields] OR CYP2E1[All Fields] OR CYP3A4[All Fields] OR CYP3A5[All Fields] OR UGT1[All Fields] OR UGT1A1[All Fields] OR UGT1A3[All Fields] OR UGT1A4[All Fields] OR UGT1A5[All Fields] OR UGT1A6[All Fields] OR UGT1A7[All Fields] OR UGT1A8[All Fields] OR UGT1A9[All Fields] OR UGT1A10[All Fields] OR UGT2[All Fields] OR UGT2A1[All Fields] OR UGT2A2[All Fields] OR UGT2A3[All Fields] OR UGT2B4[All Fields] OR UGT2B7[All Fields] OR UGT2B10[All Fields] OR UGT2B11[All Fields] OR UGT2B15[All Fields] OR UGT2B17[All Fields] OR UGT2B28[All Fields] OR B3GAT1[All Fields] OR B3GAT2[All Fields] OR B3GAT3[All Fields].

Step 4) Paste in this query into builder (using “edit”) and click “add to history”—this is referred to as “#2” in the rest of this search strategy.

“Solute Carrier Proteins”[MeSH Terms] OR “Membrane Transport Proteins”[MeSH Terms] OR “P-gp”[All Fields] OR “p-glycoprotein”[All Fields] OR BCRP[All Fields] OR OCT2[All Fields] OR “Organic Cation Transporter 2”[MeSH Terms] OR “Organic Cation Transport Proteins”[MeSH Terms] OR MATE1[All Fields] OR “SLC4A Proteins”[MeSH Terms] OR MATE-2K[All Fields] OR “SLC4A Proteins”[MeSH Terms] OR OATP[All Fields] OR OAT1[All Fields] OR “Organic Anion Transport Protein 1”[MeSH Terms] OR OAT3[All Fields] OR UGT1[All Fields] OR “Glucuronosyltransferase”[MeSH Terms] OR ABC[All Fields] OR “ATP-Binding Cassette Transporters”[MeSH Terms].

Step 5) Paste in this query into builder (using “edit”) and click “add to history”—this is referred to as “#3” in the rest of this search strategy.

(“mitragynine”[All Fields] OR “mitragynine ethanedisulfonate”[All Fields] OR “SK and F 12711”[All Fields] OR “SKF 12711”[All Fields] OR “SK and F-12711”[All Fields] OR “mitragynine, (16E)-isomer”[All Fields] OR “mitragynine, (3beta,16E)-isomer”[All Fields] OR “mmitragynine, (3beta,16E,20beta)-isomer”[All Fields] OR “kratom alkaloids”[All Fields] OR “kmitragynine monohydrochloride”[All Fields] OR “Mitragyna speciosa”[All Fields] OR “Nauclea speciose”[All Fields] OR “Biak-biak”[All Fields] OR “Cratom”[All Fields] OR “Gratom”[All Fields] OR “Ithang”[All Fields] OR “Kakuam”[All Fields] OR “Katawn”[All Fields] OR “Kedemba”[All Fields] OR “Ketum”[All Fields] OR “Krathom”[All Fields] OR “Kraton”[All Fields] OR “Kratum”[All Fields] OR “Madat”[All Fields] OR “Mambog”[All Fields] OR “Mitragynine”[All Fields] OR “Mitragynine extract”[All Fields] OR “Thang”[All Fields] OR “Thom”[All Fields] OR “7-hydroxymitragynine”[All Fields] OR “7-hydroxy-mitragynine”[All Fields] OR “mitragynine pseudoindoxyl”[All Fields] OR “Paynantheine”[All Fields]).

Step 6) Paste in this query into builder (using “edit”) and click “add to history”—this is referred to as “#4” in the rest of this search strategy.

(Pharmacokinetics[MeSH Terms] OR pharmacokinetic[All Fields]) or (inhibit[All Fields] or inhibition[All Fields]) OR substrate[All Fields].

Step 7) Paste in this query into builder (using “edit”) and click “add to history”—this is referred to as “#5” in the rest of this search strategy.

#3 AND #4 AND (#1 OR #2) AND “humans”[MeSH Terms].
